# Analysis Method and Experimental Conditions Affect Computed Circadian Phase from Melatonin Data

**DOI:** 10.1371/journal.pone.0033836

**Published:** 2012-04-12

**Authors:** Hadassa Klerman, Melissa A. St. Hilaire, Richard E. Kronauer, Joshua J. Gooley, Claude Gronfier, Joseph T. Hull, Steven W. Lockley, Nayantara Santhi, Wei Wang, Elizabeth B. Klerman

**Affiliations:** 1 Division of Sleep Medicine, Brigham and Women's Hospital, Boston, Massachusetts, United States of America; 2 Division of Sleep Medicine, Harvard Medical School, Boston, Massachusetts, United States of America; 3 Graduate School of Arts and Sciences, Harvard University, Cambridge, Massachusetts, United States of America; 4 Duke University School of Medicine, National University of Singapore, Singapore, Singapore; 5 Inserm, U846, Stem Cell and Brain Research Institute, Lyon, France; Simon Fraser University, Canada

## Abstract

Accurate determination of circadian phase is necessary for research and clinical purposes because of the influence of the master circadian pacemaker on multiple physiologic functions. Melatonin is presently the most accurate marker of the activity of the human circadian pacemaker. Current methods of analyzing the plasma melatonin rhythm can be grouped into three categories: curve-fitting, threshold-based and physiologically-based linear differential equations. To determine which method provides the most accurate assessment of circadian phase, we compared the ability to fit the data and the variability of phase estimates for seventeen different markers of melatonin phase derived from these methodological categories. We used data from three experimental conditions under which circadian rhythms - and therefore calculated melatonin phase - were expected to remain constant or progress uniformly. Melatonin profiles from older subjects and subjects with lower melatonin amplitude were less likely to be fit by all analysis methods. When circadian drift over multiple study days was algebraically removed, there were no significant differences between analysis methods of melatonin onsets (P = 0.57), but there were significant differences between those of melatonin offsets (P<0.0001). For a subset of phase assessment methods, we also examined the effects of data loss on variability of phase estimates by systematically removing data in 2-hour segments. Data loss near onset of melatonin secretion differentially affected phase estimates from the methods, with some methods incorrectly assigning phases too early while other methods assigning phases too late; missing data at other times did not affect analyses of the melatonin profile. We conclude that melatonin data set characteristics, including amplitude and completeness of data collection, differentially affect the results depending on the melatonin analysis method used.

## Introduction

Circadian phase is a major determinant of the time course and level of sleepiness, cognitive performance, many hormone concentrations and multiple other physiologic functions. Accurate measurement of circadian phase is also vital for the correct diagnosis and appropriate treatment for circadian rhythm sleep disorders. Since circadian phase of the suprachiasmatic nucleus (SCN), the site of the mammalian circadian pacemaker, cannot be measured directly in humans, outputs of the clock must be used as markers of the circadian system. Commonly used circadian phase markers include core body temperature (CBT), cortisol, and melatonin. A critical factor in choosing an appropriate marker for assessing circadian phase, either for clinical applications or research, is the influence of exogenous factors that can directly mask, or obscure, the underlying endogenous circadian rhythm of the output marker. In inpatient studies in which these exogenous factors are controlled or eliminated, melatonin-based phase assessments produce the least variable estimates of circadian phase [1] when assessed under dim light conditions. Compared to CBT and cortisol rhythms, the melatonin rhythm is less influenced by changes in sleep-wake state, exercise or mood [2], although the effects of posture remain controversial [3], [4]. Concentrations of melatonin or its metabolites can be easily obtained from blood, saliva, or urine specimens [4], [5], [6], [7].

The synthesis and daily rhythm of melatonin secretion is regulated by the SCN. In entrained individuals, melatonin levels remain low during the day with melatonin onset occurring ∼2 hours prior to bedtime, and peak near the middle of the habitual nighttime, 2 to 3 hours before the core body temperature (CBT) nadir [8]. Under non-dim light conditions, light exposure in the evening and night suppresses the endogenous onset of melatonin secretion [9]. The pathway for inhibition of melatonin secretion in response to input occurs via the retino-hypothalamic tract (RHT), the pathway through which photic information travels from the eye to the SCN. The RHT remains intact in some totally visually blind people and allows them to entrain to the 24-hour day and demonstrate melatonin suppression even in the absence of conscious vision [10], [11], [12]. Conversely, in some visually intact individuals with spinal cord injury there is no rhythmic melatonin secretion, presumably due to damage to the pathway from the SCN to the pineal gland [13], [14]. Thus, the measurement of melatonin levels can provide information on the status of the pathway from the eye to the SCN and to the pineal, as well as provide information on circadian phase and amplitude in individuals in whom this pathway is intact.

Several methods have been developed to analyze the phase and amplitude of the circadian melatonin rhythm. These methods usually calculate the ‘onset’, midpoint and ‘offset’ relative to a threshold of an assumed symmetrical melatonin secretion profile, although onset and cessation of actual synthesis are more relevant physiologically [15]. Most of these methods fall into one of three categories in which phase is determined: (i) by fitting a curve to the melatonin profile, usually a sinusoidal-based function; (ii) by determining when melatonin levels cross either an absolute concentration threshold, or an individualized threshold; or (iii) by using a physiologically-based linear differential equation model to estimate the onset of melatonin secretion, the cessation of synthesis, the amplitude of the melatonin rhythm, and rates of infusion and clearance of melatonin from the plasma or saliva.

The first two categories of methods assume that: (i) the shape of the melatonin profile is appropriate for the analysis method, including a monotonic rise; (ii) the shape of the melatonin profile does not change significantly from day-to-day or under different conditions; (iii) the amplitude of an individual's melatonin rhythm is sufficiently great to allow for curve-fitting or determination of a threshold; and (iv) the melatonin rhythm has a mainly symmetrical profile with a single peak (for pure sinusoidal-based or midpoint-based methods). There is ample evidence that such assumptions are not met in many cases. Additionally, inter-individual variations in melatonin profiles may affect phase estimates derived from some threshold-based methods (e.g. 10 pg/ml), although it is not known if the physiologically significant onset concentration may differ among individuals. Furthermore, although threshold-based methods do not require collecting the full 24-hr profile (e.g. if computing only time of crossing of 10 pg/ml), they are extremely sensitive to missing data around the threshold crossing time(s). Physiologically-based methods (e.g., linear differential equations based on the physiology such as [15]), meanwhile, can provide an estimate of pineal activity, and therefore the amplitude and rates of infusion and clearance in the plasma can be computed; these two physiologic variables cannot be determined from curve-fitting and threshold methods.

Several reports have compared different methods of analyzing melatonin, with an emphasis on the fitting of the data, rather than the use of the method in computing circadian phase. In testing their physiologically-based linear differential equation model, Brown et al. [15] compared values generated by their model with selected values from other methods, including one threshold crossing (Dim Light Melatonin Onset (DLMO) – interpolated crossing) and one 3-harmonic curve-fitting method. Van Someren and Nagtegaal [16] compared the analysis of salivary melatonin using three curve-fitting models, which included a skewed baseline cosine function, a bimodal cosine function, and a combination of these two into a bimodal skewed cosine function, with analysis from several curve-fitting and interpolating methods. These new curve-fitting models, which accounted for differences in steepness of rising and falling portions of the melatonin profile (skewed) and variations in melatonin peak (such as broader or flatter or even two peaks (bimodal)), were found not only to fit the data better than previous models but to be robust even after data points were removed to simulate missing data. They also evaluated the robustness of phase estimates in the presence of systematically added noise; this noise generated approximately 10 minutes of deviation in the phase from the original estimates. The focus of their report, however, was the fit of the data and not whether phase markers derived from different fitting methods differed significantly from one another within a subject across days or could be used in different experimental conditions, with decreased melatonin amplitude, or with different subject populations.

Previous studies assessing melatonin as a marker of human circadian phase have not addressed issues related to its reliability under different experimental circumstances. There are at least three areas of concern: (i) phase assessment methods from more than one study condition/design have not been compared to each other, raising concerns about the generalizability of the findings [1], [17]; (ii) the effects of removing data on phase assessment variability have not been systematically examined, although, in the case of missing or noisy data, fitted methods using all data points would be expected to provide more accurate results than those methods relying on interpolation, which utilize fewer points [1], [16]; and (iii) correlates of melatonin profiles that could be fit by one but not another analysis method. This report addresses all three concerns.

We compared curve-fitting, threshold, and linear differential equation mathematical model (“physiological”) methods of analyzing melatonin data. The variabilities of these methods on circadian phase estimates of data collected from multiple protocols, with different melatonin amplitudes, and from healthy but mixed populations (sighted and blind, young and older, male and female) were quantified. To test the robustness of different circadian phase assessment methods, we explored the time-dependent effects of missing data on phase estimates by removing data at different time intervals in the melatonin profile.

## Methods

### Data sets

All studies were conducted in the Brigham and Women's Hospital inpatient General Clinical Research Center or Center for Clinical Investigation, using techniques reported elsewhere (see below). All studies were approved by the Partners Healthcare Human Subjects Committee and all subjects gave written informed consent. Procedures were in compliance with U.S. Health Insurance Portability and Accountability Act regulations and the Declaration of Helsinki. All subjects were healthy according to medical history, physical exam, blood chemistries, psychological screening tests and visits with a clinical psychologist. Subjects in Studies 1–5 were not color blind; subjects in Study 6 were visually blind and some had a non-24-h sleep disorder but met all other criteria. Subjects were not using any prescription or non-prescription medications, had regular sleep/wake schedules, and had not used caffeine or tobacco for at least the week prior to entry to the inpatient portion of the study.

Three non-overlapping ∼24-h days of data were used from each subject. These three days were chosen to be under dim light conditions so that the endogenous circadian pacemaker would expect to drift uniformly at its endogenous period and therefore the variability of the markers could be tested. Complete data sets were defined as three ∼24-h days of data in which there were less than 2 consecutive hours of missing data and less than 6 total hours of missing data per 24-hr. Blood samples were drawn every 30–60 minutes in all studies. For all studies, melatonin was assayed by RIA.

#### Study 1

[18] Data were available for thirteen subjects aged 17–29 years who participated in a protocol that began with 3 baseline days (8 h of sleep and 16 h of wake) followed by a constant routine (CR, an extended period of enforced wakefulness in a semi-recumbent position with frequent small meals [19] that lasted 26–33 hr. There were then 3 days on an inverted sleep-wake schedule with 5 h of darkness centered in the middle of the inverted wake episode, a second CR, 3 more days with darkness exposure and a final CR. Light levels were 90 lux (approximately 90 lux measured at 54″ from the floor and maximum 150 lux measured at 72″ from the floor in the horizontal angle from anywhere in the room) for waking baseline days, 4 lux (approximately 3.3 lux measured at 54″ from the floor and maximum 15 lux measured at 72″ from the floor in the horizontal angle from anywhere in the room) during the CR and intervention days, and 0 lux for all sleep episodes and the darkness exposures. Data from the three CRs were used.

#### Study 2

(Santhi et al., unpublished data). Data were available from six subjects aged 19–30 years who participated in a protocol that began with 3 baseline days followed by a 50-h CR. Light levels during baseline days were 90 lux (defined as in Study 1) for baseline until the last 8 hours of the wake episode before the CR, 3 lux (approximately 1.8 lux measured at 54″ from the floor and maximum 8 lux measured at 72″ from the floor in the horizontal angle from anywhere in the room) during the last five hours of the wake episode before the CR and during the CR, and 0 lux during sleep. The three 24 hrs of data were from the 24 hours immediately before the CR and the two 24-h periods during the 50-h CR.

#### Study 3

[9], [20]. Data were available from 20 subjects aged 19–28 years who participated in a protocol that began with three baseline days followed by a 50-h CR. Light levels during the baseline day were 90 lux (defined as in Study 1) until the last 8 hours of the wake episode before the CR, 3 lux (defined as in Study 2) for the remainder of the study except for during sleep episodes when they were 0 lux. The three 24 hrs of data were from the 24 hours immediately before the CR and the two 24-h periods during the 50-h CR.

#### Study 4

[21]. Data were available from 20 healthy older subjects aged 65–81 years and 16 younger subjects aged 19–29 years who participated in a protocol that began with 3 baseline days followed by a CR. Light levels were 90 lux (defined as in Study 1) for waking baseline days, 4 lux (defined as in Study 1) during the CR and intervention days, and 0 lux for all sleep episodes. The three 24 hrs of data were from the 24 hours immediately before the CR and the two 24-h periods during the CR.

#### Study 5

[22]. Data were available from 12 subjects aged 21–32 years who participated in a protocol that included a forced desynchrony segment with 28-h “days”. Light levels were 4 lux (defined as in Study 1) during wake episodes and 0 lux during sleep episodes. Data were from the beginning of the forced desynchrony segment, with three consecutive 24-h days for all but one subject, in whom the data were from 4 consecutive 24-h days because of missing data on one of the intermediate days.

#### Study 6

[23] Data were collected from 11 visually blind (i.e., no conscious light perception) subjects, aged 27–68 years, who participated in a 38-day inpatient protocol that included a forced desynchrony segment (24 28-h “days”). Light levels were 4 lux (defined as in Study 1) during 18.67-h scheduled wake episodes and 0 lux during 9.33-h scheduled sleep episodes. Data from the first three consecutive 24-h days of the forced desynchrony segment for all but one subject, in whom the 3 data points were obtained from 4 consecutive days, due to missing data on one of the days.

Note that while the actual light levels used for these studies did not change, the descriptive terms describing light levels changed between studies conducted before and after 2004; the current nomenclature is used in this report for all studies. Further details about the lighting used in these studies are reported in the original publications.

### Melatonin analysis methods

**Figure 1:**
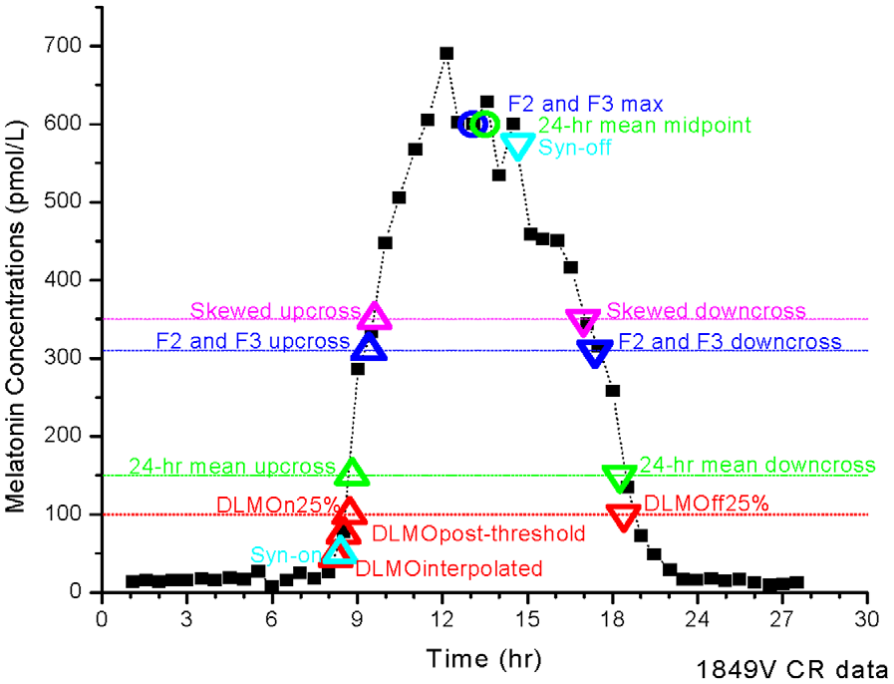
Phase Markers from Six Method Types of Analyzing Melatonin Data. Diagram of the phase markers from six methods used to analyze a single melatonin profile (subject 1849v). The upper panel includes the assayed melatonin values, plotted as melatonin concentration (pmol/L). The lower panel groups the various phase markers by method and indicates their position with respect to upper panel. Fourier-based analysis methods F(2) and F(3) have been combined in this diagram because of their similarity.

Six methods were used for the analysis of the melatonin data: two threshold-based methods, three curve fitting methods, and a physiologically-based differential equation model (Figure 1); these yielded a total of 17 circadian phase markers. In **Table 1** we provide a description of each marker with abbreviations used throughout the text.

**Table 1:** Description of melatonin phase estimate methods and abbreviations used throughout text. DLMO refers to Dim Light Melatonin Onset.

Method	Abbreviation	Number (%) of profiles not fit:		
		Young	Older	Blind
Sample time at which the first measured melatonin value exceeds the threshold of 10 pg/ml	DLMOpost-threshold	0 (0%)	6 (30%)	0 (0%)
Interpolated time when the plasma melatonin concentrations crosses the threshold of 10 pg/ml	DLMOinterpolated	0 (0%)	6 (30%)	0 (0%)
Calculated time at which melatonin levels exceed 25% of the peak of the fitted curve	DLMOn25%	3 (4%)	7 (35%)	1 (10%)
Calculated time at which melatonin levels fall below 25% of the peak of the fitted curve	DLMOff25%	3 (4%)	7 (35%)	1 (10%)
Calculated time at which melatonin levels reach the midpoint of the 24-h upcross and downcross	24hMidpoint	1 (1%)	2 (10%)	0 (0%)
Calculated time at which melatonin levels reach the 24-h mean level on the rising portion of the curve	24hUpcross	1 (1%)	2 (10%)	0 (0%)
Calculated time at which melatonin levels reach the 24-h mean level on the falling portion of the curve	24hDowncross	1 (1%)	2 (10%)	0 (0%)
Calculated time at which melatonin levels reach fit peak using a fundamental+one harmonic (F(2)) curve	F2Max	1 (1%)	2 (10%)	0 (0%)
Calculated time at which melatonin levels reach half-maximum value of F2Maxfit on rising portion of curve	F2Upcross	1 (1%)	2 (10%)	0 (0%)
Calculated time at which melatonin levels reach half-maximum value of F2Maxfit on falling portion of curve	F2Downcross	1 (1%)	2 (10%)	0 (0%)
Calculated time at which melatonin levels reach fit peak using a fundamental+two harmonic (F(3)) curve	F3Max	1 (1%)	2 (10%)	0 (0%)
Calculated time at which melatonin levels reach half-maximum value of F3Maxfit on rising portion of curve	F3Upcross	1 (1%)	2 (10%)	0 (0%)
Calculated time at which melatonin levels reach half-maximum value of F3Maxfit on falling portion of curve	F3Downcross	1 (1%)	2 (10%)	0 (0%)
Calculated time at which melatonin levels reach half of fit maximum value of the skewed bimodal cosine function on rising portion of curve	SkewedUpcross	1 (1%)	1 (5%)	4 (40%)
Calculated time at which melatonin levels reach half of fit maximum value of the skewed bimodal cosine function on falling portion of curve	SkewedDowncross	1 (1%)	1 (5%)	4 (40%)
Time of melatonin synthesis onset of melatonin as computed from the linear differential equation model	Syn-on	0 (0%)	1 (5%)	0 (0%)
Time of melatonin synthesis offset of melatonin as computed from the linear differential equation model	Syn-off	0 (0%)	1 (5%)	0 (0%)

In this analysis, “threshold-based methods” refer to methods of melatonin analysis that depend on the crossing of a pre-determined melatonin concentration. The threshold-based methods compared here included thresholds based on the dim light melatonin onset (DLMO) and from the 24-h mean of the melatonin profile. DLMO methods included (i) *DLMOinterpolated*, the time when the plasma melatonin concentrations crossed the threshold of 10 pg/ml (43.08 pmol/L) as determined by linear interpolation between the measured values flanking this value (ii) *DLMOpost-threshold*, the blood sample time at which the first measured melatonin value exceeded the 10 pg/ml threshold value [7]; and (iii) the interpolated time at which melatonin levels exceeded DLMOn 25% or fell below DLMOff 25% for at least two consecutive data points using a threshold of 25% of the fitted peak-to-trough melatonin concentrations computed using a non-orthogonal spectral analysis (NOSA) [24]. The fitted peak melatonin concentration to determine the 25% level was calculated from the melatonin profile on CR in Studies 1 to 4 (excluding first 5-h of CR) and the first forced desynchrony day (Studies 5 and 6). In total, four phase markers were derived from the DLMO methods: three onsets (DLMOinterpolated, DLMOpost-threshold, DLMOn25%) and one offset (DLMOff25%). DLMOinterpolated and DLMOpost-threshold do not require a full melatonin profile to compute. DLMOn25% and DLMOff25% require at least one 24-h profile (preferably during a constant routine condition) to determine the 25% threshold level.

Estimates based on the mean of the 24-h melatonin profile include the 24hUpcross, 24hMidpoint and 24hDowncross. For each 24-h segment of data the mean value of the melatonin concentration was computed and used as the threshold. The 24hUpcross was calculated by linear interpolation as the time at which melatonin concentration crossed this threshold value on the rising portion of the curve. The 24hDowncross was calculated in a similar fashion on the falling portion of the curve. The 24hMidpoint was calculated as the average of the 24hUpcross and 24hDowncross times.

“Curve-fitting methods” refer to methods in which a function was used to fit the entire melatonin profile; threshold-based estimates were then computed based on the fit function rather than the raw data. The curve-fitting methods used Fourier series with either 2 or 3 harmonics: F(2), a fundamental plus second harmonic (5 parameters total), and F(3), a fundamental plus second and third harmonic (7 parameters total); or a skewed bimodal cosine function [16] (6 parameters total). Circadian phase markers from each of these methods include the fit maximum (F2Max, F3Max) and the upcross (F2Upcross, F3Upcross, SkewedUpcross) and downcross (F2Downcross, F3Downcross, SkewedDowncross). The Upcross and the Downcross were computed as the interpolated values at which half the fit maximum was reached on the rising and falling portion of the curve, respectively. Data were fit using the lsqnonlin function in MatLab v.7.3 (MathWorks, Natick MA) and only data for which the model converged to a solution were included in the analysis.

The differential equation method for analyzing melatonin rhythms was originally described by Brown et al. [15] and revised by St. Hilaire and colleagues to incorporate the effect of ocular light exposure [6]. There are nine parameters that can be fit for the linear differential equation model; these were fit using the lsqnonlin function in MatLab v7.3 and only data for which the model converged to a solution were included in the analysis. Two of the markers calculated by the model were used for this analysis: Syn-on, representing the time of melatonin synthesis onset in the pineal gland, and Syn-off, for the time of synthesis cessation.

In summary, data were available from 98 subjects (67 young sighted, 20 older sighted, 11 blind). In each subject, the melatonin profiles were analyzed to generate 17 different circadian phase estimates on each of three 24-h data segments.

### Analyses

To test whether circadian phase drifted uniformly with a linear relationship across the three 24-h intervals that were assessed, we examined curvature for each subject using the 24hUpcross and DLMOinterpolated methods. To test curvature, a paired t-test was used to compare the slope of the line between phase markers on days 1 and 2, versus the slope of the line between days 2 and 3. For each of the six data sets taken from different studies, there was no curvature, which allowed us to correct for variability associated with circadian drift (see below).

Variability for each method was determined using two approaches. In the ‘With Drift’ approach, which represents the raw data analysis, we found the **standard deviation** of the clock hour of the estimate for each phase marker, assessed in each subject over the three data collection days. However, one potential source of variability between individuals in phase estimates is introduced by drift from an endogenous circadian period not equal to 24.00 (on average 24.15 h that differs between individuals [25]). To remove this potential source of variability, we first fit a line through the phase estimates computed for each marker for each subject for three consecutive days. The non-zero slope of this fit line represents the amount of drift due to (presumed) endogenous period. This fit line was subtracted from the estimates leaving the residuals of the estimates, with zero slope. The **standard error** was computed from the residuals of the estimates (i.e., standard deviation of the residuals).

The variability measure for each method was not normally distributed; the data were right skewed (with proportionally larger variability). Therefore, the data were log-transformed before all further analyses were performed to normalize the data. Linear regression models and linear mixed-effects models were used to compare different analysis methods of melatonin, with study being the random effect and age-group and sex being covariates. All tests were two-sided with alpha at the 0.05 level.

We calculated the amplitude of each melatonin curve using non-orthogonal spectral analysis (NOSA) [24], to test whether the amplitude of the melatonin rhythm affected the phase results. Amplitude was computed for the CR day (Studies 1–4) or the first forced desynchrony day (Studies 5 and 6).

We compared the goodness of fit of the different methods using Adjusted-R^2^, which accounts for the number of parameters used in fitting each model. Adjusted-R^2^ can only be calculated for curve fitting methods, such as F(2), F(3), Skewed and the linear differential equation model. An Adjusted-R^2^ value closer to 1.0 indicates a better fit.

To compare the robustness of the various methods under conditions of missing data, we selected 5 subjects at random for whom a complete data set was available and generated a series of missing data sets for each of them by removing 2 hours of consecutive data points at every 2-h time window. Four of the seventeen measures were computed for each missing data set for each subject (DLMOpost-threshold, DLMOinterpolated, F2Upcross, and Syn-on) and compared to these melatonin estimates obtained from the complete data set. These four methods were chosen because one was based on the actual sample time (DLMOpost-threshold), one was based on an interpolated threshold (DLMOinterpolated), one was based on a curve-fitting method (F2Upcross) and one was based on a differential equation method (Syn-on). These were used to determine the effect of 2 hours of missing data at different locations on the melatonin curve. The location of the data gaps within each pulse were recorded relative to the DLMOpost-threshold for each subject, computed from the complete data set. This approach normalizes the location of the data gaps for each subject to allow comparison between subjects and to identify the regions in the melatonin profile most susceptible to missing data.

## Results

Not all subjects' data could be fit with all the methods (**Table 1**). If the data could not be fit, then there was either no phase assessment produced by the analysis program because the method did not converge to a solution (for curve fitting and differential equation methods) or the phase assessment using that method was not physiologically possible. The differential equation methods had the most fits and the DLMO based methods had the fewest. There were significantly fewer fits of the data from the older subjects. The data sets that could not be fit by a method had significantly smaller amplitudes than the data sets that could be fit (63.5±57.3 (s.d.) vs. 129.7±75.5 pmol/L; p<0.001 by t-test), although there was overlap in the range of amplitudes of those that could not and those that could be fit by analysis methods (range 6.4–226.5 vs. 38.2–344.0 pmol/L, respectively).

### Variabilities

#### By study

When data were fit without first removing circadian drift, there were significant differences (P<0.05) in calculated variabilities between the six study groups using all but one melatonin method (Syn-off). Therefore, the study conditions or the different subjects in those studies affected the melatonin phase estimates when raw (with drift) phase estimates were used.

**Figure 2:**
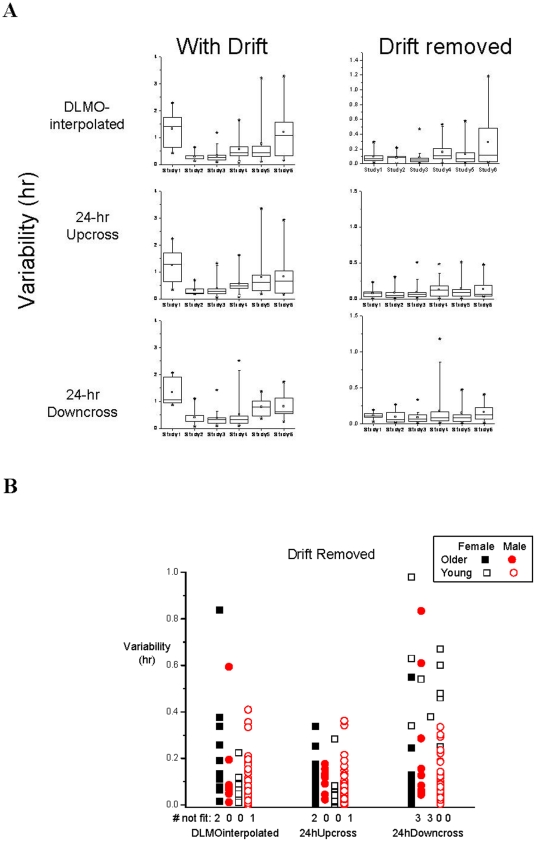
A. Variability by Study With Drift and Drift Removed. Box plots of the variability with and without drift of three circadian phase assessment methods by study. Note difference in y-axis scales between With Drift and Drift Removed. The Box plots show Maximum, Mean, Minimum, and Percentiles 99, 75, 50, 25, and 1 of the data. **Figure 2B** **. Variability by age group and sex.** Scatter plots of each subject's variability (with drift removed) of three of the methods using data from all subjects. Also indicated as (#not fit) is the number of subjects for whom that method did not yield a phase estimate.

When drift was removed, however, no significant differences in calculated variability among the six study groups were found. **Figure 2A** contains scatter plots of the variability with drift removed for three of the methods; intra-individual differences are large and the subject with the most variation in one method does not necessarily have the most variation in another method. There were statistical significant differences by age in the DLMOinterpolated method (P = 0.0297) and in the 24hDowncross method (P = 0.0445) with older subjects having higher values, and by sex in the 24hDowncross method (P = 0.0008) with female subjects having lower values in the variability for the three methods tested (**Figure 2B**); therefore to adjust for the differences among study groups, age groups and sex, study was treated as a random effect in all analyses and age group and sex were treated as covariates when the data from all the studies were combined.

#### By method

**Figure 3:**
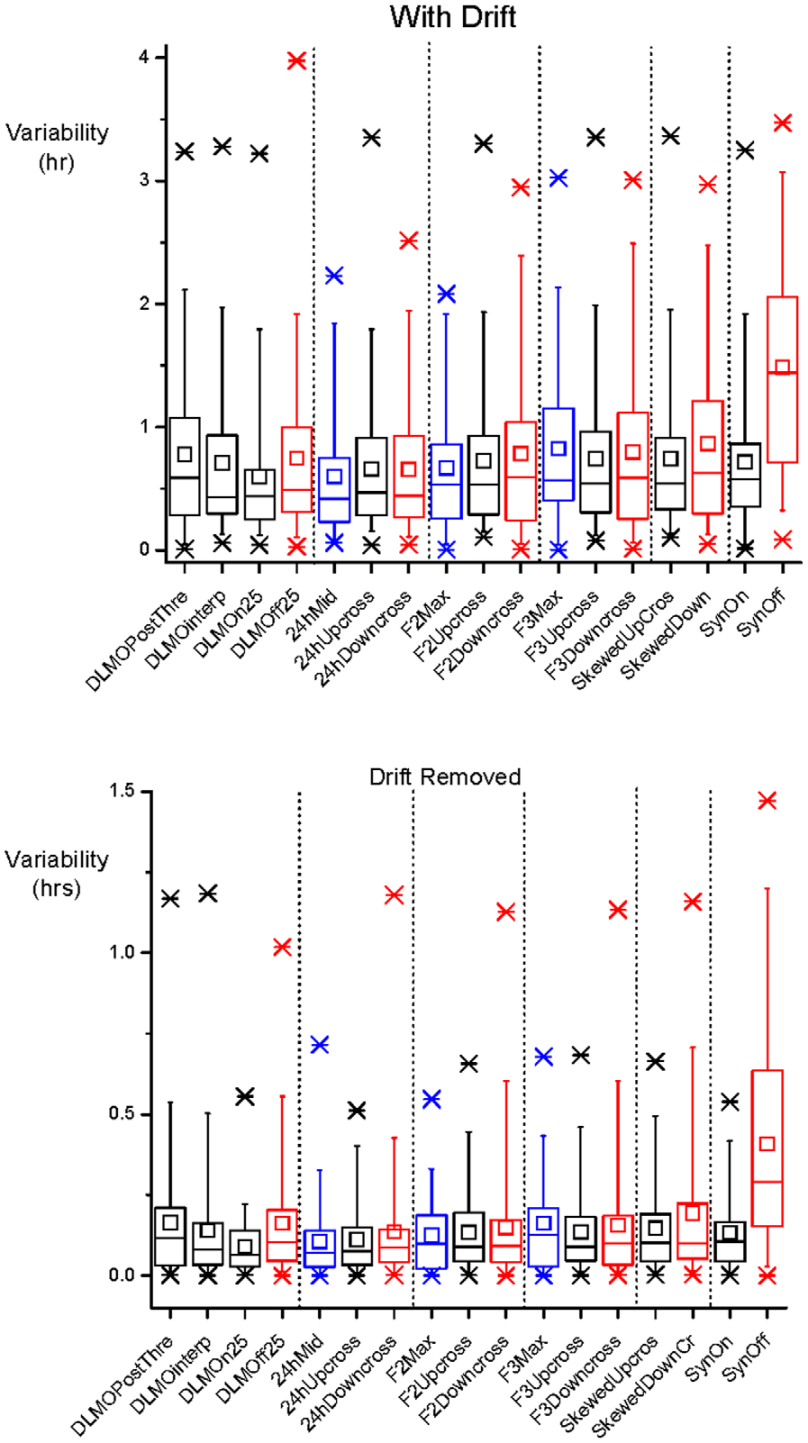
Variability of all methods With Drift and Drift Removed. The Box plots show Maximum, Mean, Minimum, and Percentiles 99, 75, 50, 25, and 1 of the data from all subjects. Black are onset methods, red are offset methods, and blue are maximum or midpoint methods. Note difference in y-axis scales between With Drift and Drift Removed.

The raw variabilities (with drift) of the 17 phase estimates differed significantly between methods (**Figure 3**, P<0.0001). The relative variability of these methods was high. Of the 17 phase estimates used, Syn-off was significantly more variable than all other methods (P<0.0001). In general, markers on the falling portion of the curve (e.g. downcross) were more variable than markers on the rising portion of the curve (e.g. upcross) (P = 0.0003).

When variability was corrected for drift (“drift removed”) across three days of data collection, individual variabilities were ∼10 times smaller than their respective raw variabilities. The variabilities of the upward/onset melatonin phase methods were not significantly different from each other (P = 0.57). The variabilities of all but 4 methods (DLMOpost_threshold, F3Downcross, SkewedUpcross and SkewedDowncross) were significantly smaller than those of the Syn-off method (P<0.05) (**Figure 3**).

There was no relationship between the fit melatonin amplitude and the DLMOn25% variability by Pearson correlation (correlation = −0.04 [N.S] for ‘With drift’ and correlation = 0.04 [N.S] with ‘Drift removed’). There was no relationship between the duration of melatonin secretion, calculated as the time between DLMOn25% and DLMOff25% and the DLMOn25% variability (Pearson correlation = −0.08 [N.S]).

### Goodness of fit and missing data

**Figure 4:**
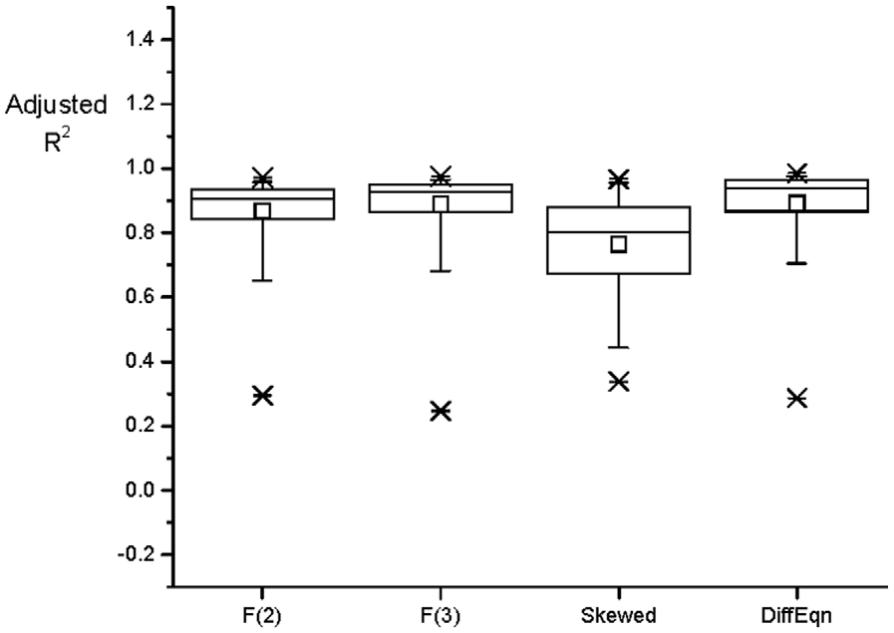
Goodness of fit using Adjusted-R^2^ for four curve-fitting methods. The Box plots show Maximum, Mean, Minimum, and Percentiles 99, 75, 50, 25, and 1 of the data from all subjects using the four curve-fitting methods: F(2), F(3), Skewed and Physiological.

There was no difference among measures when Adjusted-R^2^ was calculated for the curve-fitting methods (F(2), F(3), Skewed, and the linear differential equation model) as a measure of the goodness of fit (**Figure 4**). The outliers, with Adjusted-R^2^ values of ∼0.2, for each method were from different subjects, supporting the hypothesis that each method summarizes different aspects of each data set.

**Figure 5:**
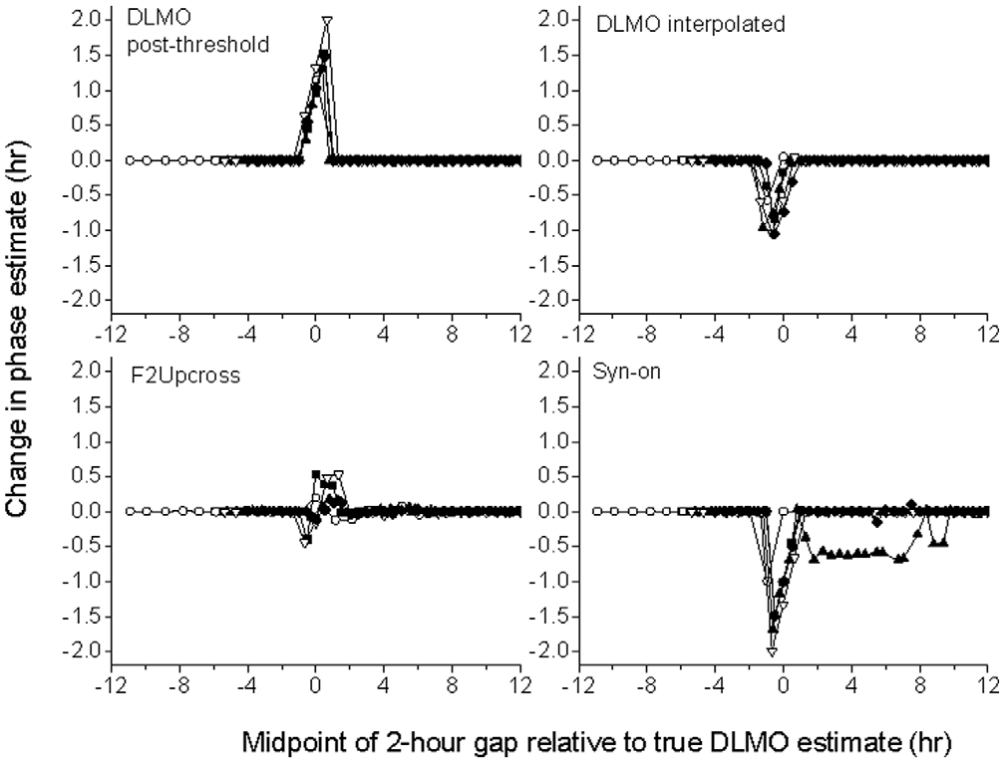
Change in phase estimates (hours) for subjects with 2-hour gaps of data. Change in phase estimates for simulations of 2-hour missing data at different times relative to the melatonin phase marker for five subjects total from Studies 1, 2 and 3. Each panel plots the change in phase estimates for a different method. Data gaps are referenced as the time of midpoint of each gap relative to the DLMOpost-threshold computed from the complete data set for each subject. Positive changes in phase estimate indicate that the estimated phase from the missing data set is later relative to the estimate from the complete data set, while negative values indicate that the estimate from the missing data set was earlier.

When points were removed from data sets for all methods, missing data had the strongest effect on phase estimates when the midpoint of the missing data fell in the ±2 hour range of the DLMOpost-threshold estimate (t = 0) from the complete data set (Figure 5). For both the Syn-on and the DLMOinterpolated values, missing data resulted in an estimated phase that was earlier (negative change in the new estimate compared to original) than the estimated phase from the complete data set in all subjects. For the DLMOpost-threshold, missing data resulted in a later estimated phase in all subjects relative to the estimate from complete data. The direction of change was variable for the F2Upcross; in most subjects, the phase estimate was earlier when missing data occurred prior to t = 0, and later when the missing data gap fell after t = 0. The magnitude of differences in phase estimates was greatest for the Syn-on estimate and for the DLMOpost-threshold estimates. In almost all cases, missing data that occurred more than two hours away from t = 0 had no effect. The exception was the phase estimate in one subject computed using Syn-on, which was affected by the missing data when the midpoint of this missing data was greater than 2 hours after t = 0. In this case, the missing data between the Syn-on and Syn-Off times affected the fit of the infusion and clearance rate parameters; because Syn-on is fit simultaneously with infusion and clearance, changes in these rates also affected the model-predicted Syn-on times.

## Discussion

Our results suggest that when there is a complete data set and the circadian drift for multi-day data is adjusted, there is no significant difference between the analysis methods used for determining melatonin secretion onset or midpoint of secretion. The studies used in this analysis minimized or eliminated many sources of noise known to affect melatonin phase estimates including light, posture, and ambient temperature [7]. Circadian drift, presumably from each individual's endogenous circadian free-running non-24-hr period, was found to be a major source of variability in melatonin phase estimates computed over consecutive days. When circadian drift is removed, methods measuring the offset of melatonin are more variable than methods measuring its onset, and therefore measures of offset should be avoided where possible. When drift is not removed, as is the case in many published studies that use melatonin phase estimates to compute circadian phase resetting, there are differences in variabilities among the methods.

Various factors such as experimental conditions, analyses methods, completeness of data (including the relative timing of the missing data) and participant characteristics (e.g. age) affected the circadian phase estimates from the melatonin data. The ability to fit the data also differed among methods, with some methods generating phase estimates for a given subject and others failing to do so. A method which is best able to fit many sources of data would be preferable, so that data sets and sources can be compared, but this study was unable to determine which method would be ideal. Researchers and clinicians should consider this evidence when choosing the method for analyzing melatonin for circadian phase information. It was assumed that other sources of variability, including those due to collection and assay, were similar across conditions.

Our finding that offset methods were more variable than onset and midpoint methods is in contrast to two previous analyses, one which found no difference in the variability of onset and offset methods [1] and one in which offset methods were found to provide more stable estimates of phase compared to onset and midpoint methods [17]. One possible explanation is the difference in study conditions between the present and former analyses. In Klerman et al. [1], all phase estimates (onsets and offsets) were measured from melatonin profiles collected during constant routine conditions in which subjects were awake in dim light. In Benloucif et al. [17], melatonin was assessed while subjects maintained a habitual sleep-wake schedule. Presumably, in this latter study, the falling portion of the curve occurred during the subject's habitual sleep episode, and therefore melatonin offsets may have been masked during sleep. In the present study, the analysis over three consecutive days included both baseline days in which subjects maintained a habitual sleep-wake schedule and constant routine days. One source of variability in the current analyses' melatonin offsets therefore may be due to unmasking of melatonin offsets during the constant routine days. This mixture of habitual sleep-wake and constant routine days would presumably not have an effect on onset variability because the rising portion of the curve is more likely to occur prior to habitual sleep onset, meaning that across three consecutive days onsets would always be assessed in dim light during wake. In addition, the physiology underlying melatonin offset is unclear and may be due to multiple factors, including the end of synthesis of melatonin and clearance of melatonin already circulating; it is difficult to mathematically determine the end of synthesis alone because these processes overlap in time.

For data sets with missing data, the location of the gap is crucial in determining the effect of the gap on phase estimate. All methods are most susceptible to gaps within two hours of the estimate determined from the complete data set, although the direction of the shift in phase estimate (earlier or later relative to the estimate obtained from the complete data set) varies between methods. Extra attention should be given to data from subjects with missing data in this range, and since the true phase is not known, appropriate adjustments cannot be made. Visual observation is crucial in verifying that the fit and marker estimates generated by the method are plausible, although such techniques are insufficient for determining the correct phase estimate. The finding that the greatest differences observed in phase shifts in response to missing data were for phase estimates of the linear differential equation model and the DLMOpost-threshold and the least for F2Upcross corroborates Klerman et al. [1], who hypothesized that curve-fitting models are more flexible than threshold-based methods. Our results show that for data sets with missing data, especially near the expected onset of melatonin secretion, curve-fitting procedures may be much better than threshold-based procedures at generating an accurate circadian phase assessment. Not all studies, however, collect melatonin samples throughout the entire secretory episode, and therefore curve-fitting methods cannot be used under these circumstances.

An analysis by Van Someren and Nagtegaal (2007) similarly found that missing data affects the phase estimate using a method in which they eliminated 4 random data points from 24 hourly samples, rather than 4 consecutive data points as was done in the present analysis. They proposed a sparse-sampling schedule with 11 of 24 data points across the 24-hr day with increased sampling clustered around expected onset and offsets; this schedule suggests that the most important sampling window for robust phase estimates of onset occurs around the expected onset, although their analysis did not systematically test this. Their analysis also does not indicate the direction of the effect on phase estimate, an effect which may be crucial when interpreting the magnitude of a phase shift as a delay or advance.

The drift in circadian phase independent of the experimental intervention (e.g., due to the intrinsic period of the circadian pacemaker) should be considered when interpreting results. The larger variability encountered when applying methods to raw data demonstrates the importance of an analysis which properly compensates for the circadian drift (which is related to intrinsic circadian period of an individual) across days. Additional, slow changes could occur in the melatonin profile parameters over multiple days that would contribute to the variability. The calculation used in this study to estimate variability while accounting for drift is relatively simple, but it requires three or more days of data. In the case of isolated or missing data, it is impossible to use this analysis to compensate properly for the drift. Unfortunately, the variabilities for the method cannot be converted to variability for a single sample because the current analyses were based on methods and not on individuals. Therefore, we can not calculate the statistical error in the melatonin phase estimate of a single subject for a single day for the different methods.

The choice of method or marker for an analysis of melatonin data depends on many factors. Our analysis suggests that the most robust phase estimates will be obtained using any of the methods looking at melatonin estimates on the rising portion of the curve provided that at least 3 complete melatonin profiles are collected over consecutive days in dim light in order to adjust for drift; Syn-on has the additional advantage of being relevant physiologically. When these conditions cannot be met to remove drift, some methods may be more accurate depending on the amount of data that can be collected.

Our analysis did not systematically evaluate the robustness of the measures in the presence of noise other than that due to intrinsic drift. An analysis by Van Someren and Nagtegaal (2007) reported the robustness of each phase estimate to added noise by multiplying each data point by a variable randomly selected between 0.8 and 1.2. Thus, although we know which methods are more robust in the presence of this noise, we do not know the form of this noise. Our analysis instead assumes that such noise is already present in the data due to collection or assay; noise due to other factors such as light, posture and ambient temperature was minimized in most of the studies used here, but not necessarily in other facilities. We do not know, however, the statistical distribution of this noise and we assume that the statistical distribution is consistent across days within a subject and consistent across subjects.

Our analyses suggest that all precautions should be taken to prevent missing data in the 2-hour window before and after expected onset. It is impossible to know *a priori* where the onset of melatonin will occur, particularly if the experimental protocol includes an intervention that alters circadian phase, and therefore at least one complete melatonin profile is necessary to establish the window during which melatonin samples should be taken in order to avoid errors in phase estimates due to poor sampling. This is especially true in the clinical setting in which patients with circadian rhythm disorders may have melatonin onsets occurring outside of the expected window.

As no differences were found in the variability of phase estimates in subjects with a low amplitude melatonin rhythm, we hypothesize that our findings will be applicable for salivary melatonin samples, which have lower concentrations than plasma samples, although saliva is rarely collected uninterrupted for multiple days. The ability of a method to fit the data, especially if the data are low amplitude or have missing samples, is also important. Since melatonin phase is used for diagnosis and treatment of circadian disorders and for timing experimental interventions, the choice of analysis method may potentially influence results. Therefore, careful consideration of the subject characteristics, the study conditions and the ability to collect complete data sets are vital.

Melatonin can be an accurate marker of human circadian pacemaker phase with appropriate application of analysis methods to datasets and experimental conditions. Even under such conditions, in the presence of missing data or low amplitude rhythms, the choice of melatonin analysis method may affect the result obtained. Variability in phase estimates was significantly lower when drift associated with free-running circadian rhythms was removed and when a complete melatonin profile was available. Given that such conditions may not be always available, comparison of markers revealed that those based on the detection of a time during rising melatonin concentrations were similar in their variability and therefore are recommended for use.
